# Silencing Mediated by the *Schizosaccharomyces pombe* HIRA Complex Is Dependent upon the Hpc2-Like Protein, Hip4

**DOI:** 10.1371/journal.pone.0013488

**Published:** 2010-10-18

**Authors:** Holly E. Anderson, Alexander Kagansky, Josephine Wardle, Juri Rappsilber, Robin C. Allshire, Simon K. Whitehall

**Affiliations:** 1 Institute for Cell and Molecular Biosciences, Newcastle University, Newcastle, United Kingdom; 2 Wellcome Trust Centre for Cell Biology and Institute of Cell Biology, University of Edinburgh, Edinburgh, Scotland, United Kingdom; Texas A&M University, United States of America

## Abstract

**Background:**

HIRA (or Hir) proteins are conserved histone chaperones that function in multi-subunit complexes to mediate replication-independent nucleosome assembly. We have previously demonstrated that the *Schizosaccharomyces pombe* HIRA proteins, Hip1 and Slm9, form a complex with a TPR repeat protein called Hip3. Here we have identified a new subunit of this complex.

**Methodology/Principal Findings:**

To identify proteins that interact with the HIRA complex, rapid affinity purifications of Slm9 were performed. Multiple components of the chaperonin containing TCP-1 complex (CCT) and the 19S subunit of the proteasome reproducibly co-purified with Slm9, suggesting that HIRA interacts with these complexes. Slm9 was also found to interact with a previously uncharacterised protein (SPBC947.08c), that we called Hip4. Hip4 contains a HRD domain which is a characteristic of the budding yeast and human HIRA/Hir-binding proteins, Hpc2 and UBN1. Co-precipitation experiments revealed that Hip4 is stably associated with all of the other components of the HIRA complex and deletion of *hip4^+^* resulted in the characteristic phenotypes of cells lacking HIRA function, such as temperature sensitivity, an elongated cell morphology and hypersensitivity to the spindle poison, thiabendazole. Moreover, loss of Hip4 function alleviated the heterochromatic silencing of reporter genes located in the mating type locus and centromeres and was associated with increased levels of non-coding transcripts derived from centromeric repeat sequences. Hip4 was also found to be required for the distinct form of silencing that controls the expression of *Tf2* LTR retrotransposons.

**Conclusions/Significance:**

Overall, these results indicate that Hip4 is an integral component of the HIRA complex that is required for transcriptional silencing at multiple loci.

## Introduction

The assembly of specific regions of eukaryotic genomes into heterochromatin is important for processes such as gene silencing, nuclear organisation, development, dosage compensation and chromosome segregation. In the fission yeast, *S. pombe* the assembly of heterochromatin is directed by the RNAi machinery [1], [2], [3]. Heterochromatic domains, such as those at centromeres, contain repeat sequences that are transcribed at low levels during S phase [3], [4]. The resulting non-coding transcripts are processed to form siRNAs that are loaded into an RNAi effector complex called RITS [5], which directs the recruitment of a complex containing the histone H3 lysine 9 (H3K9) methyltransferase, Clr4 [1], [2], [3]. In turn, methylated H3K9 is bound by chromodomain proteins such as the HP1 homolog, Swi6 [6]. The integrity of heterochromatin at centromeres is also dependent upon the HIRA chromatin assembly factors, Hip1 and Slm9 [7]. Mutation of either protein alleviates the silencing of marker genes located in centromeric outer repeats and leads to increased levels of chromosome missegregation [7].

HIRA (or Hir) proteins are evolutionarily conserved histone chaperones that mediate nucleosome assembly independently of DNA replication [8], [9], [10]. Accordingly, human HIRA is found associated with histone H3.3, which is deposited into chromatin outside of S phase [11], [12]. Roles for HIRA proteins in the assembly and maintenance of heterochromatin have been reported in a range of organisms. In *S. cerevisiae* the mutation of *HIR* genes does not alleviate silencing but does exacerbate the telomeric and *HM* silencing defects associated with inactivation of chromatin assembly factor 1 (CAF-1) [13], [14], [15]. In *S. pombe* the loss of HIRA proteins results in silencing defects at centromeres and the mating type (*mat*) locus even in the presence of functional CAF-1 [7], [16]. In human fibroblasts, HIRA drives the assembly of senescence associated heterochromatin in association with another histone chaperone, ASF1a [17], [18] and in *Arabidopsis* HIRA is a component of a complex that maintains *knox* gene silencing in developing leaves [19], [20].

HIRA proteins execute their functions in the context of multi-subunit complexes. Human HIRA is associated with multiple proteins [12] and *S. cerevisiae* Hir1 and Hir2 are found to be stably associated with two structurally unrelated proteins, Hir3 and Hpc2 (also called Hir4) [8], [9]. Similarly, the fission yeast HIRA proteins, Hip1 and Slm9, interact with Hip3, a homolog of *S. cerevisiae* Hir3 [16]. In this study we report the identification and characterisation of an *S. pombe* Hpc2-related protein that we have called Hip4. Co-precipitation experiments indicate that Hip4 is stably associated with all of the subunits of the HIRA complex (Hip1, Slm9 and Hip3) and a *hip4*Δ strain has defects that are characteristic of cells lacking HIRA function. Deletion of *hip4^+^* also alleviates heterochromatic transcriptional silencing at the *mat* locus and centromeres, and de-represses the expression of *Tf2* LTR retrotransposons. Thus our data indicate that Hip4 is an integral component of the HIRA complex.

## Results

**Figure 1:**
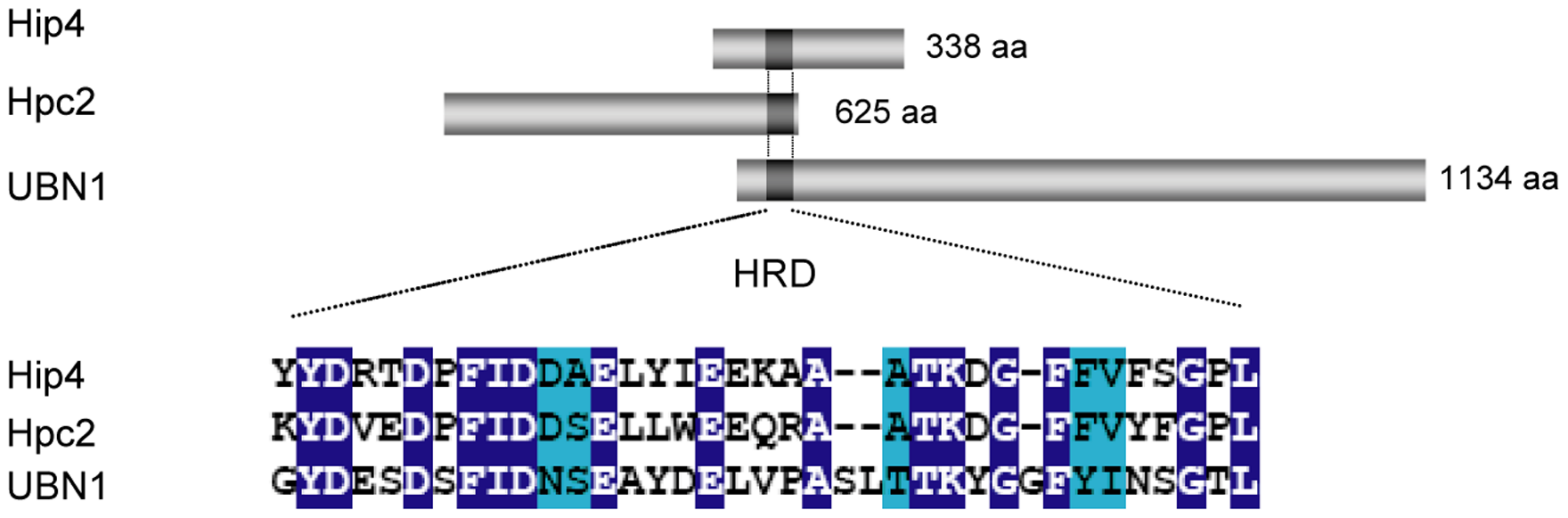
An Hpc2/UBN1 related protein in fission yeast. A schematic diagram showing Hip4 (SPBC947.08c), *S. cerevisiae* Hpc2 and human UBN1. The conserved HRD domain is shaded dark grey. Shown below is a sequence alignment of the HRD domain. Identical residues are shaded blue and similar residues are shaded turquoise.

**Figure 2:**
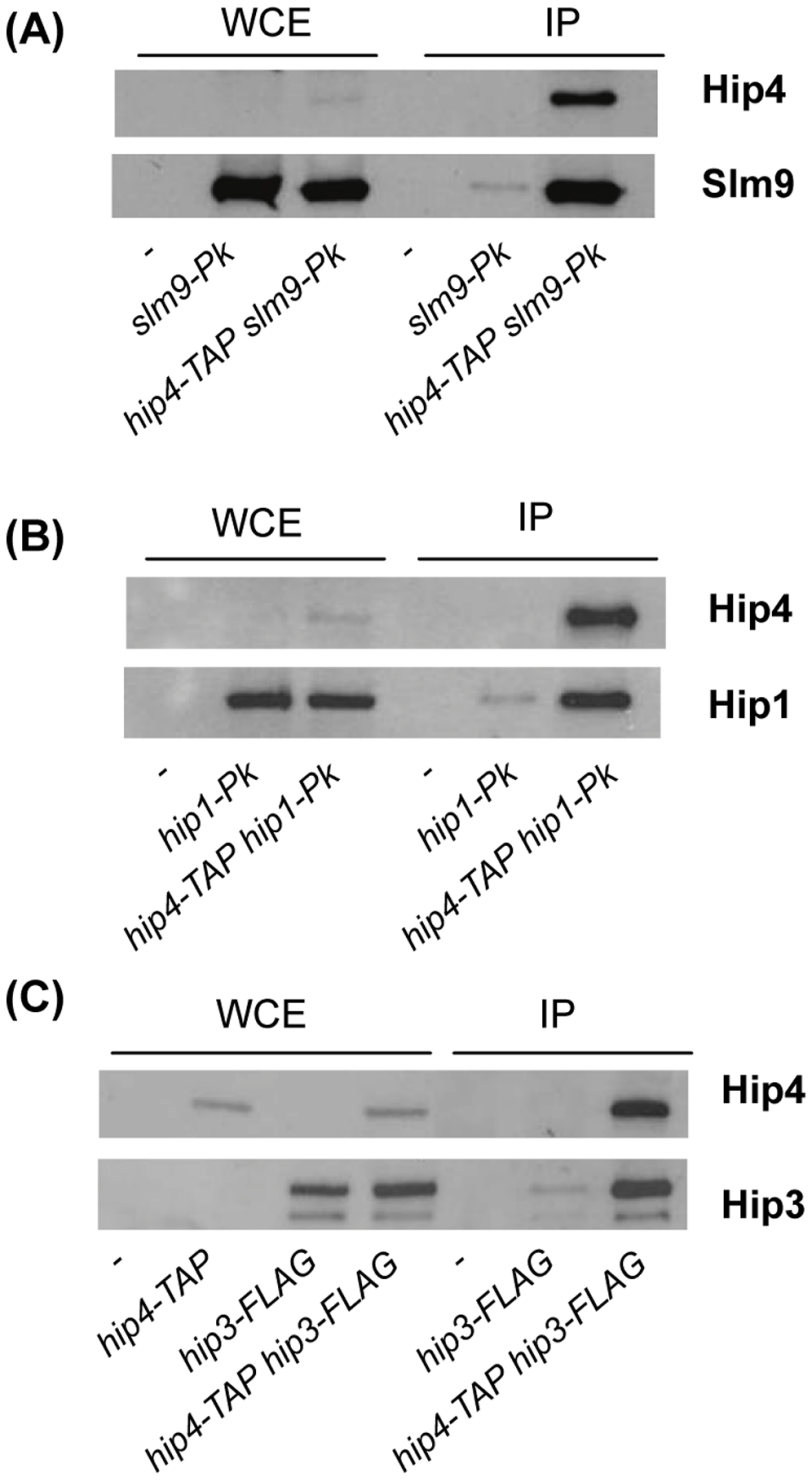
Hip4 is stably associated with the subunits of the HIRA complex. (**A**) Whole cell extracts (WCE) or whole cell extracts partially purified with IgG-coupled Dynal beads (IP) were analysed by western blotting with anti-Pk and peroxidase anti-peroxidase (α-PAP) antibodies. The strains used are indicated below the lane. (**B**) As for (A). (**C**) As for A except that anti-FLAG antibody was used in place of anti-Pk antibody. Experiments were performed at least twice and a representative example is shown.

The fission yeast HIRA complex contains Hip1, Slm9 and a structurally unrelated protein called Hip3 [16]. In order to identify additional components of the HIRA complex, and to identify other factors that interact with this complex, we employed a rapid affinity purification protocol that facilitates the isolation of large protein assemblies [21]. Extracts from cells expressing a Slm9-FLAG fusion, were affinity purified and the resulting proteins were identified by liquid chromatography-tandem mass spectrometry. Numerous proteins were found to reproducibly co-purify with Slm9-FLAG (Table 1). Multiple components of the chaperonin containing TCP-1 complex (CCT, also called TRiC) [22] were present in Slm9-FLAG purifications. CCT is responsible for the folding of numerous proteins [23] and this suggests that it is required for the biogenesis of the HIRA complex. This analysis also suggests that HIRA interacts with the 19S regulatory particle of the proteasome [24] because multiple ATPase subunits (Rpt1, Rpt2, Rpt3, Rpt4 and Rpt5) from this complex co-purified with Slm9. Analysis of the list of Slm9-interacting proteins also revealed SPBC947.08c, a previously uncharacterised protein that we named Hip4 for HIRA interacting protein 4. Hip4 has limited homology to *S. cerevisiae* Hpc2 and human UBN1; Hpc2 is a component of the *S. cerevisiae* HIR complex [8], [9] and during the course of this study Banumathy and co-workers reported that UBN1 functions in conjunction with human HIRA [25]. Although the overall level of sequence identity of SPBC947.08c to both Hpc2 and UBN1 is low, it does contain a short sequence motif, termed an Hpc2-related domain (HRD), which is a characteristic of these proteins (Fig 1). In order to determine whether Hip4 also interacts with the other components of the HIRA complex its chromosomal locus was tagged with the TAP epitope (*hip4-TAP*). Whole cell extracts from *hip4-TAP* cells were partially purified using IgG magnetic beads and analysed by western blotting. This confirmed that Slm9 co-purifies with Hip4 (Fig 2A) and furthermore, similar experiments revealed that both Hip1 and Hip3 co-precipitate with Hip4 (Fig 2B & C). Therefore, Hip4 is stably associated with all the components of the *S. pombe* HIRA complex.

**Table 1:** Slm9 co-purifying proteins.

Gene	Peptides	Score^a^	Protein^b^
*hip1^+^*	49	2750	HIRA protein
*slm9^+^*	39	1851	HIRA protein
*hip3^+^*	33	1680	HIRA interacting protein 3
*ura1^+^*	15	682	carbamoyl-phosphate synthase
*SPBC947.08c*	9	455	Hpc2-related protein
*ths1^+^*	7	276	threonine-tRNA ligase
*hsp90^+^*	6	324	Hsp90 chaperone
*cts1^+^*	5	160	CTP synthase
*rpt2^+^*	4	140	19S proteasome regulatory subunit
*rpt3^+^*	4	180	19S proteasome regulatory subunit
*anc1^+^*	4	206	adenine nucleotide carrier
*lys4^+^*	4	168	homocitrate synthase
*cct1^+^*	4	162	chaperonin-containing T-complex alpha subunit
*cct8^+^*	4	134	chaperonin-containing T-complex theta subunit
*dfr1^+^*	3	144	dihydrofolate reductase
*sir1^+^*	3	128	sulfite reductase beta subunit
*nda3^+^*	3	129	tubulin beta
*ade1^+^*	3	115	phosphoribosylamine-glycine ligase
*rad24^+^*	3	169	14-3-3 protein
*gpd1^+^*	3	124	glycerol-3-phosphate dehydrogenase
*tef3^+^*	3	136	translation elongation factor
*cdc48^+^*	3	107	AAA family ATPase
*ilv1^+^*	3	163	acetolactate synthase catalytic subunit
*pss1^+^*	3	136	Hsp70 protein
*mts4^+^*	3	131	19S proteasome regulatory subunit
*vma2^+^*	2	111	V-type ATPase V1 subunit B
*tub1^+^*	2	75	tubulin alpha 2
*SPBC365.16*	2	60	sequence orphan
*rpt5^+^*	2	63	19S proteasome regulatory subunit
*atp2^+^*	2	53	F1-ATPase beta subunit
*vrs2^+^*	2	102	valine-tRNA ligase
*fba1^+^*	2	135	fructose-bisphosphate aldolase
*SPAC17A5.15c*	2	71	glutamate-tRNA ligase
*rvb1^+^*	2	69	AAA family ATPase Rvb1
*cct6^+^*	2	105	chaperonin-containing T-complex zeta subunit
*SPCC1827.06c*	2	98	aspartate semialdehyde dehydrogenase
*pgk1^+^*	2	61	phosphoglycerate kinase
*cct4^+^*	2	77	chaperonin-containing T-complex delta subunit
*rpt4^+^*	2	84	19S proteasome regulatory subunit
*ssa1^+^*	2	96	Hsp70 protein
*rpt1^+^*	2	113	19S proteasome regulatory subunit

^*a*^MASCOT Score.

^*b*^The descriptions of the protein functions are based on information for *S. pombe* in GeneDB (http://www.genedb.org/genedb/pombe/index.jsp).

**Figure 3:**
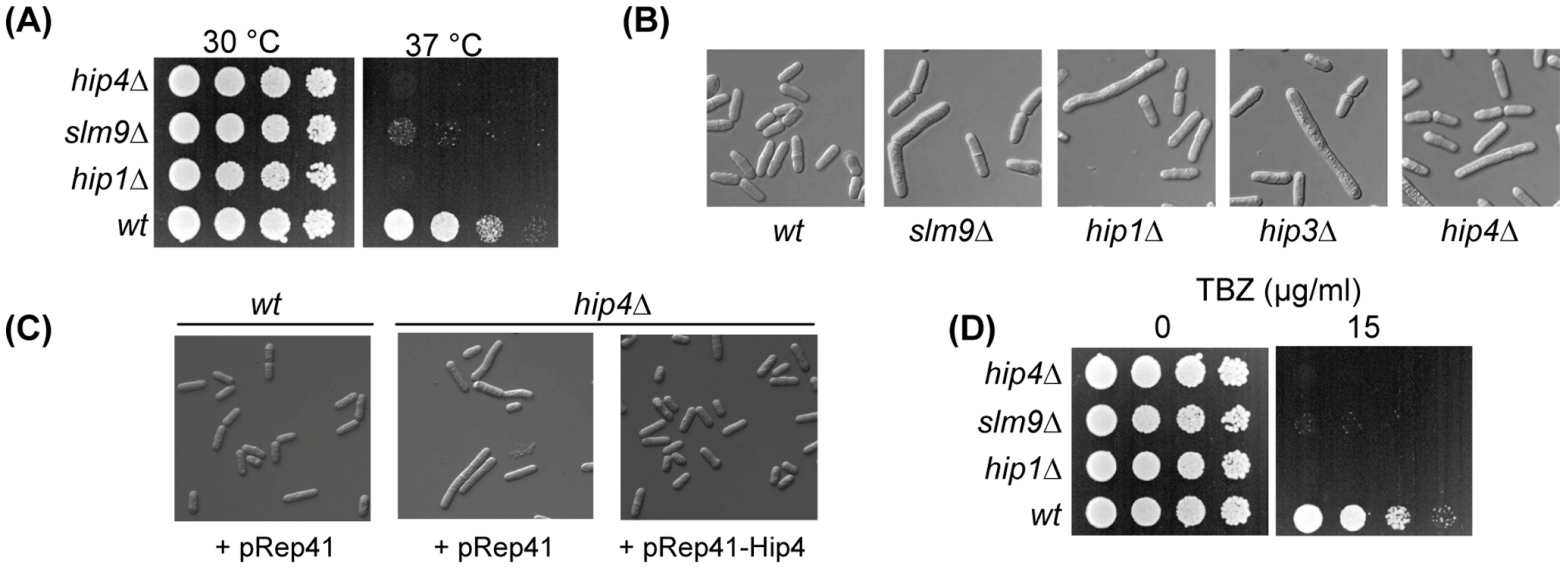
Phenotypes associated with deletion of *hip4* ^+^. (**A**) *hip4*Δ cells are temperature sensitive. The indicated strains were grown to exponential phase, subjected to five-fold serial dilutions and spotted onto YE5S agar plates and incubated at 30°C or 37°C. (**B**) Comparison of the morphology of wild type, *hip1*Δ, *slm9*Δ, *hip3*Δ and *hip4*Δ cells. (**C**) Comparison of the morphology of wild type cells transformed with empty vector (pRep41), *hip4*Δ cells transformed with empty vector and *hip4*Δ cells transformed with a plasmid expressing *hip4^+^* (pRep41-Hip4) from the *nmt* promoter. (**D**) *hip4*Δ cells are hypersensitive to spindle damage. The indicated strains were grown to exponential phase, subjected to five-fold serial dilutions and spotted onto YE5S agar or YE5S supplemented with thiabendazole (TBZ) at 15 µg/ml. Plates were incubated at 30°C. Experiments were performed at least three times and a representative example is shown.

In order to investigate the function of this protein we constructed a *hip4Δ* strain. Cells lacking Hip4 were found to be viable but exhibited a number of phenotypes that are reminiscent of cells lacking the function of the HIRA complex (Fig 3). Like other HIRA mutants, the *hip4Δ* strain was temperature sensitive showing a limited ability to proliferate at 37°C (Fig 3A). Furthermore, microscopic examination of *hip4Δ* cells revealed that they had an elongated phenotype similar to *hip1Δ*, *slm9Δ* and *hip3Δ* cells (Fig 3B), which are known to have a G2 cell cycle delay [7], [16], [26]. The introduction of a plasmid ectopically expressing *hip4* (pRep41Pk-Hip4) rescued the elongated cell phenotype of *hip4*Δ cells (Fig 3C) confirming that this defect is due to lack of Hip4 function.

**Figure 4:**
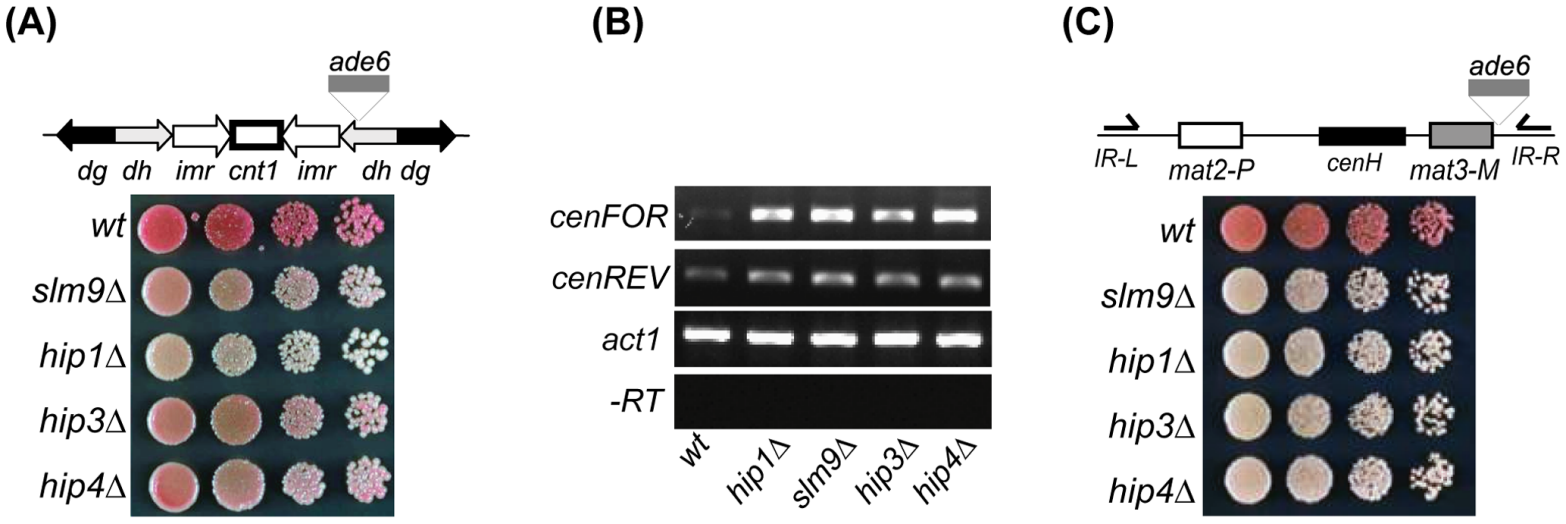
Loss of Hip4 impairs transcriptional silencing. (**A**) Centromeric (*dh-dg*) repeat silencing. Strains containing the *otrR1::ade6^+^* allele in combination with the appropriate deletions were grown to log phase in YE5S medium subjected to 5-fold serial dilutions and spotted on to YE5S agar lacking adenine. Plates were incubated for 3–4 days at 30°C. (**B**) Centromeric transcripts accumulate in HIRA mutants. RNA purified from the indicated strains was subjected to strand-specific RT-PCR analysis. One primer complementary to the cenFOR or cenREV transcript was included for the reverse transcription step and the second primer was added during the PCR amplification step. Control reactions omitting the reverse transcription step (-RT) were included to demonstrate the absence of DNA. (**C**) Strains containing the *mat3-M::ade6^+^* allele in combination with the appropriate deletions were treated as described for (A). Experiments were performed at least twice and a representative example is shown.

The HIRA complex is required for accurate chromosome segregation and consistent with this *hip1Δ*, *slm9Δ* and *hip3Δ* cells exhibit hypersensitivity to thiabendazole (TBZ), a drug which depolymerises microtubules and thus impairs the function of the mitotic spindle [7], [16]. Deletion of *hip4^+^* also resulted in a marked increase in TBZ sensitivity, suggesting that Hip4 is also required for accurate chromosome segregation (Fig 3D). Increased sensitivity to TBZ is often correlated with dysfunctional centromeric heterochromatin. In fission yeast, centromeric heterochromatin is assembled onto arrays of *dg*-*dh* repeats that flank a central core domain [27]. Marker genes inserted into *dg*-*dh* repeats are subjected to strong transcriptional silencing and so cells carrying the *ade6^+^* gene in this region form dark red colonies when adenine is limiting [28] (Fig 4A). Deletion of *hip1^+^*, *slm9^+^* or *hip3^+^* results in reduced *trans*-gene silencing and the formation of pink colonies [7], [16]. Similarly, introduction of the *hip4Δ* allele into this background resulted in the formation of pink colonies indicating that Hip4 is also required for reporter gene silencing at *dg-dh* repeats (Fig 4A).

Paradoxically, the proper assembly of ‘silent’ centromeric heterochromatin requires transcription of the *dg-dh* repeats from both strands [1], [2], [3]. These non-coding transcripts are processed by the RNAi machinery to form siRNAs which play an important role in the assembly of heterochromatin at the centromere. Transcription the *dg-dh* repeats is differentially regulated: the reverse strand (cenREV) is transcribed in wild type cells at low levels and rapidly processed by the RNAi machinery whereas transcription in the forward direction (cenFOR) is limited by heterochromatin [29]. Accordingly, mutations that impair heterochromatin function often result in increased levels of cenFOR transcripts. Quantitative RT-PCR analysis revealed that transcripts from *dg-dh* repeats were increased (1.59 fold [±0.1]) in a *slm9*Δ background. We therefore used strand-specific RT-PCR to further analyse *dg-dh* transcripts and found that increased levels of cenFOR transcripts were detectable in *hip1Δ*, *slm9Δ*, *hip3Δ* and *hip4Δ* cells (Fig 4B). This is consistent with a role for the HIRA complex in the function of pericentric heterochromatin.

Heterochromatin is also found at the *mat* locus, although at this region the assembly of heterochromatin is only partially dependent upon the RNAi machinery [30]. Marker genes that are inserted into the *mat* region are subjected to strong transcriptional silencing that is dependent upon the HIRA complex [7], [16] (see also Fig 4C). Deletion of *hip4^+^* also abolished the silencing of an *ade6^+^* reporter located at *mat3-M* (Fig 4C). This indicates that the function of the HIRA complex at heterochromatin requires the Hip4 subunit.

**Figure 5:**
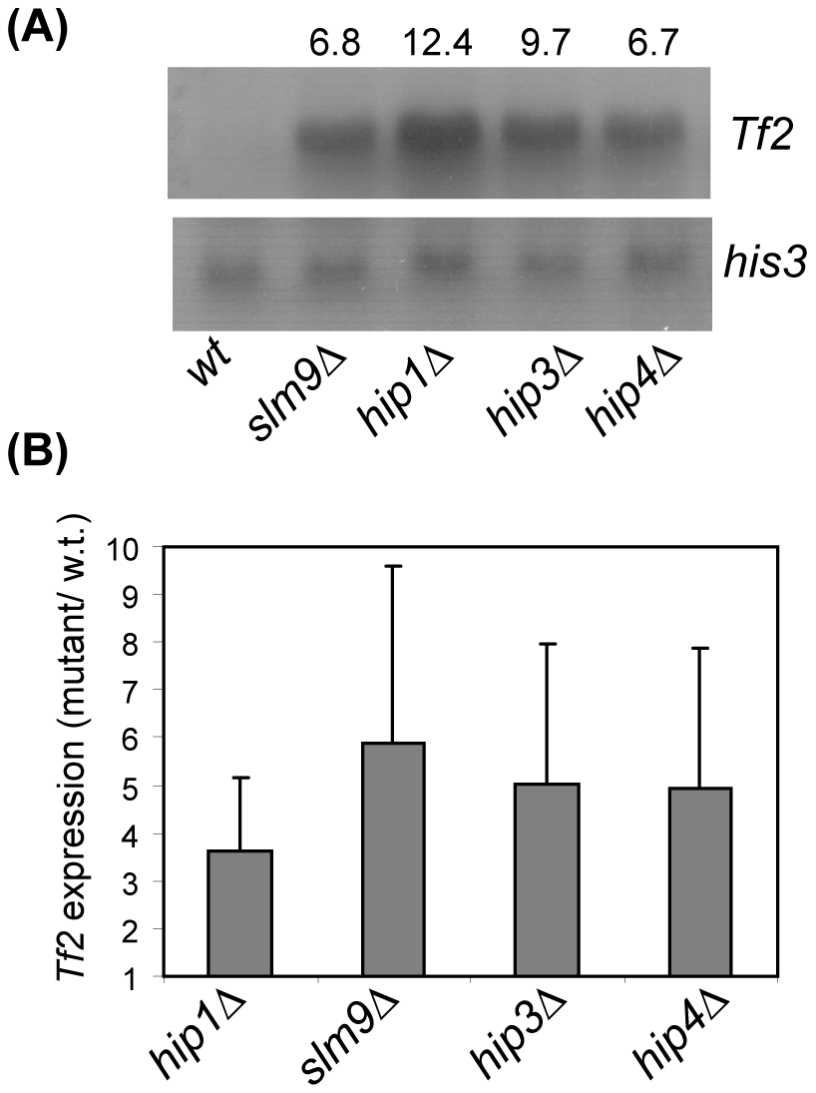
Hip4 represses the expression of *Tf2* retrotransposons. (**A**) Total RNA was prepared from the indicated strains and subjected to northern blotting with the indicated probes with *his3^+^* mRNA serving as a loading control. The fold increase in *Tf2* mRNA levels relative to wild type cells was determined by Phosphor Imager analysis and is indicated above the lanes. (**B**) *Tf2* mRNA levels were determined in the indicated strains using quantitative (real-time) RT-PCR analysis. Data are the mean of three experiments and error bars indicate ± SD.

The genome of the sequenced *S. pombe* strain (972), harbours 13 full-length copies of the *Tf2* LTR retrotransposon [31]. These elements are silenced by a mechanism that is distinct from the *mat* locus and centromeres being independent of H3K9 methylation [16], [32]. Instead the transcriptional silencing of these elements requires CENP-B homologues [33] and multiple histone deacetylases (Clr6, Clr3 and Hst4) [32], [33], [34]. It is also dependent upon the function of the HIRA complex as global *Tf2* mRNA levels are dramatically increased in *hip1*Δ, *slm9*Δ and *hip3*Δ backgrounds [16]. Furthermore, northern blotting and quantitative RT-PCR analyses revealed that *Tf2* mRNA levels were similarly increased in *hip4Δ* cells (Fig 5A & B), indicating that LTR retrotransposon silencing requires Hip4. Taken together our data indicate that Hip4 is an integral component of the HIRA complex that is required for silencing at multiple loci.

## Discussion

Here we have identified a new component of the *S. pombe* HIRA complex. Co-precipitation analyses indicate that Hip4 is stably associated with Hip1, Slm9 and Hip3. Furthermore, deletion of *hip4^+^* results in phenotypes that are characteristic of cells lacking HIRA complex function. Hip4 is necessary for the integrity of heterochromatin at centromeres and at the *mat* locus and is also required for the distinct form of silencing that represses the expression of *Tf2* LTR retrotransposons.

Hip4 appears to be the fission yeast counterpart of *S. cerevisiae* Hpc2 and human UBN1, however the sequence similarity between these proteins is essentially restricted to the HRD domain (also called HUN, for Hpc2-Ubinuclein-1 domain) [35]. The HRD domain of UBN1 binds to the N-terminal WD repeats of HIRA and similarly, the HRD domain is required for the interaction of Hpc2 with *S. cerevisiae* Hir1 [25]. Furthermore, a human HIRA mutant that harbours an arginine to lysine substitution in amino acid 227, between the fourth and fifth WD repeats, is unable to bind to UBN1 [25]. This critical arginine residue is conserved in *S. pombe* Hip1, and indeed the whole WD repeat region of Hip1 and human HIRA exhibit considerable sequence similarity [7]. It is therefore highly likely that Hip4 directly interacts with the WD repeats of Hip1. However, Slm9 also contains recognisable WD repeat motifs in its N-terminal region and so a direct interaction between Hip4 and Slm9 cannot be discounted.

**Figure 6:**
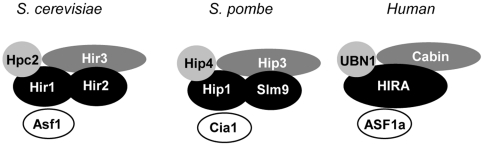
The composition of HIRA/Hir Complexes. A schematic diagram comparing the components of the *S. cerevisiae*, *S. pombe* and Human HIRA/Hir complexes. HIRA/Hir subunits are shaded black, Hir3 orthologs are shaded dark grey, Hpc2 orthologs are shaded light grey and the Asf1 orthologs are white.

The identification of Hip4 indicates that the composition of the *S. pombe* HIRA complex is highly similar to that of budding yeast, in which the HIRA/Hir subunits interact with two structurally unrelated proteins (Hir3 and Hpc2 in *S. cerevisiae*; Hip3 and Hip4 in *S. pombe*). Moreover, recent evidence indicates that this fundamental degree of similarity extends to human HIRA complexes. Following the identification of UBN1 as an ortholog of Hpc2 [25], recent bioinformatic analyses have suggested that the human counterpart of Hir3/Hip3 is the calcineurin binding protein, Cabin1 [35]. Importantly, both UBN1 and Cabin1 are known to be associated with HIRA because a histone H3.3 complex was found to be composed of HIRA, UBN1, Cabin and another histone chaperone ASF1a [12]. Human HIRA interacts with ASF1a through its B domain, which is located in central portion of the protein [36] and an interaction between *S. cerevisiae* Hir1 and Asf1 has also been demonstrated [15]. While affinity purifications of fission yeast HIRA proteins have not identified the *S. pombe* Asf1 homologue (Cia1), an interaction between this protein and the B-domain from Hip1 has been demonstrated *in vitro* [37]. Thus the core interactions of the budding yeast, fission yeast and human HIRA/Hir proteins appear to be highly conserved (Fig 6).

The affinity purification experiments also indicate an interaction between the HIRA complex and the proteasome and so it is possible that HIRA subunit levels may be regulated by proteasomal degradation. However, alternative explanations for this interaction are possible given that Slm9 co-purifies with 19S ATPase subunits that have previously been linked to chromatin and transcriptional regulation. Indeed, it has been proposed that 19S ATPase subunits can exist independently of the proteasome and exert non-proteolytic functions in transcriptional control [38]. In *S. cerevisiae* the 19S ATPases, Sug1 and Sug2, which are counterparts of *S. pombe* Rpt6 and Rpt4, are recruited to the promoters of specific genes (*HSP26*, *HSP104* and *GAD1*) and are required for their induction in response to stress [39]. Similarly, in HeLa cells 19S ATPase subunits regulate chromatin modifications and expression at the CTIIA and MHC-II promoters [40]. In both cases the function of the proteasomal subunits is independent of proteolysis [39], [40]. Furthermore, others have shown that the proteasome co-operates with the histone chaperone FACT in the disassembly of nucleosomes at *PHO5* during transcriptional induction [41]. It is therefore possible that 19S ATPase subunits antagonise the repressive functions of the HIRA complex.

Slm9 affinity purifications also revealed interactions with subunits of the chaperonin CCT, which is known to mediate the folding of numerous proteins [23]. This suggests that the biogenesis of HIRA complexes may be CCT-dependent. In support of this hypothesis, analysis of CCT substrates in *S. cerevisiae* has revealed several chromatin remodelling complexes and furthermore CCT is known to have a high affinity for the β-propeller fold adopted by WD repeats [22]. The functional relationship between HIRA and many other Slm9 co-purifying proteins is obscure. For example, enzymes involved in nucleotide and amino acid metabolism (Ura1, Cts1, Lys4 Ade1 and Anc1) and also tRNA synthetases (Vas2, Ths1 and SPAC17A5.15c) were reproducibly found in Slm9-immunoprecipitates. However, an interaction between HIRA and CCT may go some way to explaining the presence of these unexpected proteins. The CCT interaction network in *S. cerevisiae* is known to include numerous enzymes [23] and therefore, many proteins found in Slm9 pull-downs may be present by virtue of being CCT substrates.

Our data indicate that Hip4, like all other components of the HIRA complex, is required for heterochromatic silencing. Similarly, UBN1 is required for the HIRA/ASF1a pathway that establishes domains of senescence associated heterochromatin in senescent fibroblasts [25]. However, the functions that the HRD domain proteins fulfil within HIRA/Hir complexes are not understood. Although, it has been proposed that HRD domain proteins function as histone chaperones that bind to histone tails [35], there is as yet no direct evidence to support this. Interestingly, analysis of *S. cerevisiae* Hpc2 has revealed that it exhibits genetic interactions that are not observed with Hir1, Hir2 and Hir3 [35], [42], suggesting that it has functions that are independent of the Hir complex. Given that it is now clear that HRD domain proteins have been conserved throughout evolution [25], [35], it will be important to determine the precise roles of these proteins in the organization of chromatin structure.

## Materials and Methods

### Plasmids and Strains

The strains used in this study are described in Table 2. The *hip4^+^* ORF was disrupted using one-step gene replacement. A fragment from the 5′ end of the *hip4^+^* ORF was PCR amplified using oligonucleotides Hip4KOA (5′-GATGCTCTTCTTGTTCGTACTCGTTTCAAAG -3′) and Hip4KOB (5′-CAGTATCTCCTTAAGCTTGACGAACAAAAGCGAAGTATAG-3′) and a fragment from the 3′ end of the *hip4^+^* ORF was PCR amplified using oligonucleotides Hip4KOC (5′-TTTGTTGCTAAGCTTAAGGAGATACTGTGAAAATCG-3′) and Hip4KOD (5′-ATCGATTGACAGATCTTCAGG-3′). These fragments were used as the template in an overlapping PCR reaction with oligonucleotides primers Hip4KOA and Hip4KOD. The resulting fragment was cloned into pGEM-T (Promega) to yield pGEM-Hip4 and the 1.8 kb *ura4^+^* cassette from pRep42 was then cloned into the *Hind*III site to give plasmid pGEM-Hip4::ura4^+^. The *Pst*I-*Bgl*II fragment from pGEM-Hip4::ura4^+^ was used to transform a diploid strain and integration at the correct locus was confirmed by PCR. The genomic locus of *hip4^+^* was tagged with the TAP epitope by PCR amplifying a fragment using the oligonucleotides primers hip4Pk-PstI (5′-GTTCACCTGCAGGAAGGAGATACTGTGAAAATC-3′) and hip4Pk-BamHI (5′-CAGTTCGGATCCTGGGATGCTACTAGTTGGAAT-3′). The fragment was digested with *Bam*HI and *Pst*I and cloned into the *Pst*I and *Bam*HI sites of pRip42-CTAP [16]. The resulting plasmid was linearised with *Bst*XI and transformed into the appropriate strains. The pRep41Pk-Hip4 plasmid was constructed by PCR amplifying the *hip4^+^* ORF using oligonucleotide primers hip4NdeI (5′-GTCACGCATATGTCGCTTTGTCTGGCGAC -3′) and hip4Pk-BamHI, digesting the resulting DNA fragment with *Bam*HI and *Nde*I and cloning it into pRep41PkN [43]. The genomic locus of *slm9^+^* was tagged with three copies of the FLAG epitope (3XFLAG) by PCR amplifying a fragment from p3X-FLAG-CMV (Sigma) using oligonucleotides 3XFLAG3′ (5′-GCTAGCTCCATGGATCACTACTTGTCATCGTC-3′) and 3XFLAG5′ (5′-CGTAGGCGGATCCCCGGGTGACTACAAAGACCATGACGGT-3′). The resulting fragment was digested with *Bam*HI and *Nco*I and cloned into the *Bam*HI and *Nco*I sites of pRip42-Slm9-CTAP to give plasmid pRip42-Slm9-3XFLAG. The resulting plasmid was digested with *Xba*I and transformed into wild type cells.

**Table 2:** Strains used in this study.

Strain	Genotype	Source
NT5	*h^-^ ade6-216 leu1-32 ura4-D18*	Lab stock
NT4	*h^+^ ade6-210 leu1-32 ura4-D18*	Lab stock
SW281	*h^+^ ade6-210 leu1-32 ura4-D18 slm9-3XFLAG(ura4^+^)*	This study
SW288	*h^-^ ade6-M210 leu1-32 ura4-D18 hip1::ura4^+^*	[7]
SW318	*h^-^ ade6-M210 leu1-32 ura4-D18 hip3::ura4^+^*	[16]
SW409	*h^-^ ade6-M210 leu1-32 ura4-D18 hip4::ura4^+^*	This study
JK2246	*h^-^ leu1-32 ura4-D18 slm9::ura4^+^*	[26]
SW248	*h^+^ ade6-210 leu1-32 ura4-D18 slm9-Pk(ura4^+^)*	[7]
SW442	*h^-^ ade6-210 leu1-32 ura4-D18 slm9-Pk(ura4^+^) hip4-TAP(ura4^+^)*	This study
SW376	*h^-^ ade6-216 leu1-32 ura4-D18 hip3-3XFLAG(LEU2)*	[16]
HA45	*h^-^ ade6-216 leu1-32 ura4-D18 hip4-TAP(ura4^+^)*	This study
SW594	*h^-^ ade6-216 leu1-32 ura4-D18 hip3-3XFLAG(LEU2) hip4-TAP(ura4^+^)*	This study
SW187	*h^+^ ade6-210 leu1-32 ura4-D18 hip1-Pk(ura4^+^)*	[7]
HA84	*h^-^ ade6-216 leu1-32 ura4-D18 hip1-Pk(ura4^+^) hip4-TAP(ura4^+^)*	This study
FY1182	*h^+^ ade6-M210 leu1-32 ura4-D18 otr1*R*(Sph*I*)::ade6^+^*	[28]
SW118	*h^+^ ade6-M210 leu1-32 ura4-D18 slm9::ura4^+^ otr1*R*(Sph*I*)::ade6^+^*	[7]
SW151	*h^+^ ade6-M210 leu1-32 ura4-D18 hip1::ura4^+^ otr1*R*(Sph*I*)::ade6^+^*	[7]
SW364	*h^+^ ade6-M210 leu1-32 ura4-D18 hip3::ura4^+^ otr1*R*(Sph*I*)::ade6^+^*	[16]
SW440	*h^-^ ade6-M210 leu1-32 ura4-D18 hip4::ura4^+^ otr1*R*(Sph*I*)::ade6^+^*	This study
PG1672	*mat1-PΔ17::LEU2 mat3-M(Eco*RV*)::ade6 ade6-M210 leu1-32 ura4-D18*	[49]
SW149	*mat1-PΔ17::LEU2 mat3-M(Eco*RV*)::ade6 ade6-M210 leu1-32 ura4-D18 slm9::ura4^+^*	[7]
SW150	*mat1-PΔ17::LEU2 mat3-M(Eco*RV*)::ade6 ade6-M210 leu1-32 ura4-D18 hip1::ura4^+^*	[7]
SW588	*mat1-PΔ17::LEU2 mat3-M(Eco*RV*)::ade6 ade6-M210 leu1-32 ura4-D18 hip4::ura4*	This study

### Large scale immunoaffinity purification

Immunoaffinity purifications for LC-MS/MS analysis were performed as described [21], with the following modifications. Purifications were performed on extracts derived from 5 g of cells using Dynabeads coupled to anti-FLAG M2 antibody (Sigma, F1804) for 15 mins. The immunoprecipitated material was treated with 500 U of Benzonase, washed, subjected to on-bead tryptic digestion, and prepared for LC-MS/MS analysis as described previously [44]. Briefly, immunoprecipitated material was subjected to on-bead Tryptic digestion. The resulting peptides were desalted using StageTips [45], [46] and analyzed using LC-MS on a LTQ-Orbitrap (Thermo Fisher Scientific) coupled to HPLC via a nanoelectrospray ion source. The six most intense ions of a full MS acquired in the orbitrap analyzer were fragmented and analyzed in the linear ion trap. The MS data were analyzed using MaxQuant [47] and proteins identified by searching MS and MS/MS data using the MASCOT search engine (Matrix Science, UK). Table 1 lists proteins identified in at least two independent purifications from cells expressing the Slm9-FLAG fusion and absent from four independent control purifications from untagged cells. Ribosomal proteins (common contaminants) were also excluded.

### Co-immunoprecipitations

Whole cell extracts were prepared as described previously except that the standard lysis buffer was substituted with HB buffer (25mM Tris-HCl [pH 7.5], 15 mM EGTA, 15 mM MgCl_2_, 0.1% NP40, 1 mM DTT, 0.1 mM NaF). Immunoprecipitations were performed by adding 25 µL IgG-coupled Dynal beads to 1 mg of whole protein extract and incubating for 1 hour at 4°C with gentle agitation. Beads were recovered and washed three times with 1 ml HB buffer. Samples were electrophoresed through SDS-PAGE gels and subjected to western blotting using monoclonal anti-FLAG antibodies (Sigma), monoclonal anti-Pk (Serotec) and Peroxidase Anti-Peroxidase soluble complex produced in rabbit (Sigma).

### Northern Blotting

RNA was purified and analysed as described previously [7]. Gene specific probes were produced by PCR amplification from genomic DNA using the appropriate primers. All probes were labelled with α-[^32^P]-dCTP using a Prime-a-Gene labelling kit (Promega).

### RT-PCR

For strand-specific RT-PCR, RNA was purified as previously described [48] and subjected to RT-PCR using a One Step RT-PCR kit (Qiagen). One primer complementary to the sense or antisense transcript was added during first strand cDNA synthesis while the second primer was added prior to the PCR amplification steps. cDNA for quantitative (real-time) RT-PCR was made using a Superscript II kit (Invitrogen). Real-time PCR reactions were performed using a LightCycler 2.0 PCR system (Roche) and SYBR Green mix (Molecular Probes) using the appropriate primers [48]. Reactions were normalised using primers specific to *act1^+^*. Data for quantitative (real-time) RT-PCR are the mean of three experiments. Error bars indicate ± SD.

## References

[1] Buhler M, Moazed D (2007). Transcription and RNAi in heterochromatic gene silencing.. Nat Struct Mol Biol.

[2] Grewal SI, Jia S (2007). Heterochromatin revisited.. Nat Rev Genet.

[3] Kloc A, Martienssen R (2008). RNAi, heterochromatin and the cell cycle.. Trends Genet.

[4] Chen ES, Zhang K, Nicolas E, Cam HP, Zofall M (2008). Cell cycle control of centromeric repeat transcription and heterochromatin assembly.. Nature.

[5] Verdel A, Jia S, Gerber S, Sugiyama T, Gygi S (2004). RNAi-mediated targeting of heterochromatin by the RITS complex.. Science.

[6] Bannister AJ, Zegerman P, Partridge JF, Miska EA, Thomas JO (2001). Selective recognition of methylated lysine 9 on histone H3 by the HP1 chromo domain.. Nature.

[7] Blackwell C, Martin KA, Greenall A, Pidoux A, Allshire RC (2004). The *Schizosaccharomyces pombe* HIRA-like protein Hip1 is required for the periodic expression of histone genes and contributes to the function of complex centromeres.. Mol Cell Biol.

[8] Green EM, Antczak AJ, Bailey AO, Franco AA, Wu KJ (2005). Replication-independent histone deposition by the HIR complex and Asf1.. Curr Biol.

[9] Prochasson P, Florens L, Swanson SK, Washburn MP, Workman JL (2005). The HIR corepressor complex binds to nucleosomes generating a distinct protein/DNA complex resistant to remodeling by SWI/SNF.. Genes Dev.

[10] Ray-Gallet D, Quivy JP, Scamps C, Martini EM, Lipinski M (2002). HIRA is critical for a nucleosome assembly pathway independent of DNA synthesis.. Mol Cell.

[11] Ahmad K, Henikoff S (2002). The histone variant H3.3 marks active chromatin by replication-independent nucleosome assembly.. Mol Cell.

[12] Tagami H, Ray-Gallet D, Almouzni G, Nakatani Y (2004). Histone H3.1 and H3.3 complexes mediate nucleosome assembly pathways dependent or independent of DNA synthesis.. Cell.

[13] Kaufman PD, Cohen JL, Osley MA (1998). Hir proteins are required for position-dependent gene silencing in *Saccharomyces cerevisiae* in the absence of chromatin assembly factor I.. Mol Cell Biol.

[14] Qian Z, Huang H, Hong JY, Burck CL, Johnston SD (1998). Yeast Ty1 retrotransposition is stimulated by a synergistic interaction between mutations in chromatin assembly factor I and histone regulatory proteins.. Mol Cell Biol.

[15] Sharp JA, Fouts ET, Krawitz DC, Kaufman PD (2001). Yeast histone deposition protein Asf1p requires Hir proteins and PCNA for heterochromatic silencing.. Curr Biol.

[16] Greenall A, Williams ES, Martin KA, Palmer JM, Gray J (2006). Hip3 interacts with the HIRA proteins Hip1 and Slm9 and is required for transcriptional silencing and accurate chromosome segregation.. J Biol Chem.

[17] Zhang R, Chen W, Adams PD (2007). Molecular dissection of formation of senescence-associated heterochromatin foci.. Mol Cell Biol.

[18] Zhang R, Poustovoitov MV, Ye X, Santos HA, Chen W (2005). Formation of MacroH2A-containing senescence-associated heterochromatin foci and senescence driven by ASF1a and HIRA.. Dev Cell.

[19] Guo M, Thomas J, Collins G, Timmermans MC (2008). Direct repression of KNOX loci by the ASYMMETRIC LEAVES1 complex of Arabidopsis.. Plant Cell.

[20] Phelps-Durr TL, Thomas J, Vahab P, Timmermans MC (2005). Maize rough sheath2 and its *Arabidopsis* orthologue ASYMMETRIC LEAVES1 interact with HIRA, a predicted histone chaperone, to maintain *knox* gene silencing and determinacy during organogenesis.. Plant Cell.

[21] Oeffinger M, Wei KE, Rogers R, DeGrasse JA, Chait BT (2007). Comprehensive analysis of diverse ribonucleoprotein complexes.. Nat Methods.

[22] Spiess C, Meyer AS, Reissmann S, Frydman J (2004). Mechanism of the eukaryotic chaperonin: protein folding in the chamber of secrets.. Trends Cell Biol.

[23] Dekker C, Stirling PC, McCormack EA, Filmore H, Paul A (2008). The interaction network of the chaperonin CCT.. EMBO J.

[24] Hanna J, Finley D (2007). A proteasome for all occasions.. FEBS Lett.

[25] Banumathy G, Somaiah N, Zhang R, Tang Y, Hoffmann J (2009). Human UBN1 is an ortholog of yeast Hpc2p and has an essential role in the HIRA/ASF1a chromatin-remodeling pathway in senescent cells.. Mol Cell Biol.

[26] Kanoh J, Russell P (2000). Slm9, a novel nuclear protein involved in mitotic control in fission yeast.. Genetics.

[27] Pidoux AL, Allshire RC (2004). Kinetochore and heterochromatin domains of the fission yeast centromere.. Chromosome Res.

[28] Ekwall K, Cranston G, Allshire RC (1999). Fission yeast mutants that alleviate transcriptional silencing in centromeric flanking repeats and disrupt chromosome segregation.. Genetics.

[29] Volpe TA, Kidner C, Hall IM, Teng G, Grewal SI (2002). Regulation of heterochromatic silencing and histone H3 lysine-9 methylation by RNAi.. Science.

[30] Jia S, Noma K, Grewal SI (2004). RNAi-independent heterochromatin nucleation by the stress-activated ATF/CREB family proteins.. Science.

[31] Bowen NJ, Jordan IK, Epstein JA, Wood V, Levin HL (2003). Retrotransposons and their recognition of pol II promoters: a comprehensive survey of the transposable elements from the complete genome sequence of *Schizosaccharomyces pombe*.. Genome Res.

[32] Hansen KR, Burns G, Mata J, Volpe TA, Martienssen RA (2005). Global effects on gene expression in fission yeast by silencing and RNA interference machineries.. Mol Cell Biol.

[33] Cam HP, Noma K, Ebina H, Levin HL, Grewal SI (2008). Host genome surveillance for retrotransposons by transposon-derived proteins.. Nature.

[34] Durand-Dubief M, Sinha I, Fagerstrom-Billai F, Bonilla C, Wright A (2007). Specific functions for the fission yeast Sirtuins Hst2 and Hst4 in gene regulation and retrotransposon silencing.. EMBO J.

[35] Balaji S, Iyer LM, Aravind L (2009). HPC2 and ubinuclein define a novel family of histone chaperones conserved throughout eukaryotes.. Mol Biosyst.

[36] Tang Y, Poustovoitov MV, Zhao K, Garfinkel M, Canutescu A (2006). Structure of a human ASF1a-HIRA complex and insights into specificity of histone chaperone complex assembly.. Nat Struct Mol Biol.

[37] Malay AD, Umehara T, Matsubara-Malay K, Padmanabhan B, Yokoyama S (2008). Crystal structures of fission yeast histone chaperone Asf1 complexed with the Hip1 B-domain or the Cac2 C terminus.. J Biol Chem.

[38] Gonzalez F, Delahodde A, Kodadek T, Johnston SA (2002). Recruitment of a 19S proteasome subcomplex to an activated promoter.. Science.

[39] Sulahian R, Sikder D, Johnston SA, Kodadek T (2006). The proteasomal ATPase complex is required for stress-induced transcription in yeast.. Nucleic Acids Res.

[40] Koues OI, Mehta NT, Truax AD, Dudley RK, Brooks JK (2010). Roles for common MLL/COMPASS subunits and the 19S proteasome in regulating CIITA pIV and MHC class II gene expression and promoter methylation.. Epigenetics Chromatin.

[41] Ransom M, Williams SK, Dechassa ML, Das C, Linger J (2009). FACT and the proteasome promote promoter chromatin disassembly and transcriptional initiation.. J Biol Chem.

[42] Collins SR, Miller KM, Maas NL, Roguev A, Fillingham J (2007). Functional dissection of protein complexes involved in yeast chromosome biology using a genetic interaction map.. Nature.

[43] Craven RA, Griffiths DJ, Sheldrick KS, Randall RE, Hagan IM (1998). Vectors for the expression of tagged proteins in *Schizosaccharomyces pombe*.. Gene.

[44] Bayne EH, Portoso M, Kagansky A, Kos-Braun IC, Urano T (2008). Splicing factors facilitate RNAi-directed silencing in fission yeast.. Science.

[45] Rappsilber J, Ishihama Y, Mann M (2003). Stop and go extraction tips for matrix-assisted laser desorption/ionization, nanoelectrospray, and LC/MS sample pretreatment in proteomics.. Anal Chem.

[46] Rappsilber J, Mann M, Ishihama Y (2007). Protocol for micro-purification, enrichment, pre-fractionation and storage of peptides for proteomics using StageTips.. Nat Protoc.

[47] Cox J, Mann M (2008). MaxQuant enables high peptide identification rates, individualized p.p.b.-range mass accuracies and proteome-wide protein quantification.. Nat Biotechnol.

[48] Anderson HE, Wardle J, Korkut SV, Murton HE, Lopez-Maury L (2009). The fission yeast HIRA histone chaperone is required for promoter silencing and the suppression of cryptic antisense transcripts.. Mol Cell Biol.

[49] Thon G, Verhein-Hansen J (2000). Four chromo-domain proteins of *Schizosaccharomyces pombe* differentially repress transcription at various chromosomal locations.. Genetics.

